# Multiple liver metastases of pulmonary carcinoid successfully treated by two-stage hepatectomy combined with embolization of portal vein branches: Report of a case

**DOI:** 10.1016/j.ijscr.2020.05.043

**Published:** 2020-05-29

**Authors:** Teppei Kamada, Shinji Onda, Yuki Takano, Taro Sakamoto, Ryo Kikuchi, Katsuhiko Yanaga

**Affiliations:** a Department of Surgery, The Jikei University School of Medicine, 3-25-8 Nishi-shimbashi, Minato-ku, Tokyo, 105-8461, Japan; b Department of Pathology, The Jikei University School of Medicine, 3-25-8 Nishi-shimbashi, Minato-ku, Tokyo, 105-8461, Japan

**Keywords:** AC, atypical pulmonary carcinoid, CT, computed tomography, ENETS, European Neuroendocrine Tumor Society, FLRV, future liver remnant volume, Gd-EOB-DTPA, gadolinium ethoxybenzyl diethylenetriamine pentaacetic acid, GEP-NET, gastroenteropancreatic neuroendocrine tumor, MRI, magnetic resonance imaging, PET, positron emission tomography, TC, typical carcinoid, Pulmonary carcinoid, Liver metastases, Two-stage hepatectomy, Portal vein embolization

## Abstract

**Introduction:**

The prognosis of atypical pulmonary carcinoid with liver metastases is extremely poor, and patients with multiple liver metastases are often treated using non-surgical therapies. We report a case with multiple liver metastases from atypical pulmonary carcinoid that was successfully treated using two-stage hepatectomy combined with embolization of portal vein branches.

**Presentation of case:**

A 48-year-old man was referred to our department after multiple liver tumors were detected in both liver lobes on computed tomography. He had undergone right upper lobectomy of the lung for atypical pulmonary carcinoid (T2a, N0, M0; Stage IB) 2 years previously. Positron emission tomography-computed tomography showed no extrahepatic tumor manifestations. The tumors were located in segment 2, 3, 5/8 and the right hepatic vein drainage area. We planned complete resection of metastases in a two-stage hepatectomy. The first stage comprised concomitant left lateral segmentectomy, partial hepatectomy of segment 5/8 and portal vein embolization of the posterior segmental branches. The second stage comprised resection of the right hepatic vein drainage area, performed 21 days after the first surgery. Histopathological diagnosis was liver metastases of atypical pulmonary carcinoid. Postoperative bile leak developed, which was treated with endoscopic retrograde biliary drainage and percutaneous bile leak drainage. He has been followed for 24 months postoperatively without tumor recurrence.

**Discussion:**

Two-stage hepatectomy may represent an option for bilobar multiple liver metastases from atypical pulmonary carcinoid.

**Conclusion:**

We successfully treated a patient with multiple liver metastases of atypical pulmonary carcinoid using a two-stage hepatectomy combined with portal vein embolization of the posterior segmental branches.

## Highlights

• The prognosis of atypical pulmonary carcinoid with liver metastases is poor, and the patients are often treated using non-surgical therapies.
• We successfully treated multiple liver metastases from atypical pulmonary carcinoid by using two-stage hepatectomy combined with embolization of portal vein branches.
• Two-stage hepatectomy may be a good option for bilobar multiple liver metastases of atypical pulmonary carcinoid.

## 1 Introduction

Atypical pulmonary carcinoid (AC) is a more aggressive variant of a peripheral pulmonary carcinoid tumor.

The prognosis of AC with liver metastases is extremely poor. Patients with multiple liver metastases from AC are often treated using non-surgical therapies such as somatostatin analogue, chemotherapy, peptide receptor radionuclide therapy, radiofrequency ablation or transarterial embolization [1,2]. Two-stage hepatectomy has recently been reported as a safe procedure that improves outcomes bilobar multiple colorectal liver metastases [3,4]. However, few reports have described hepatectomy for bilobar multiple liver metastases of AC. We report a case with bilobar liver metastases from AC that was successfully treated using two-stage hepatectomy, which is reported in line with the SCARE criteria [5].

## 2 Case presentation

**Fig. 1:**
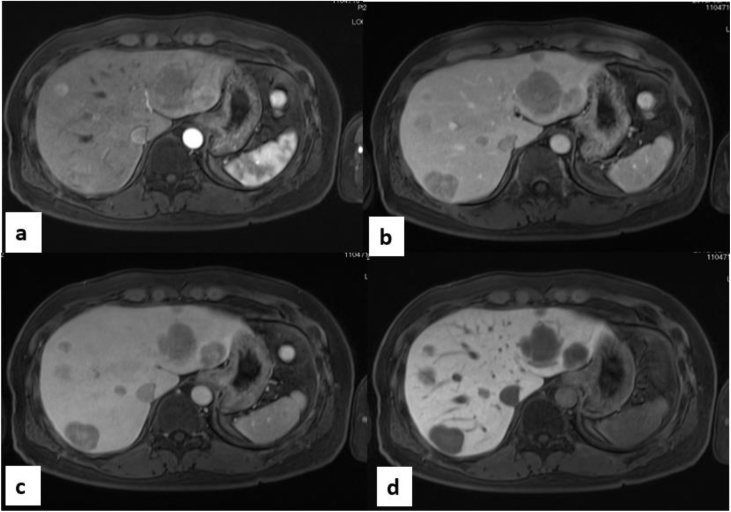
Gd-EOB-DTPA-MRI imaging. Gd-EOB-DTPA-MRI demonstrated multiple metastases (11 metastases) in both liver lobes (arrows). (a) arterial phase (b) portal phase (c) equilibrium phase (d) hepatobiliary phase

**Fig. 2:**
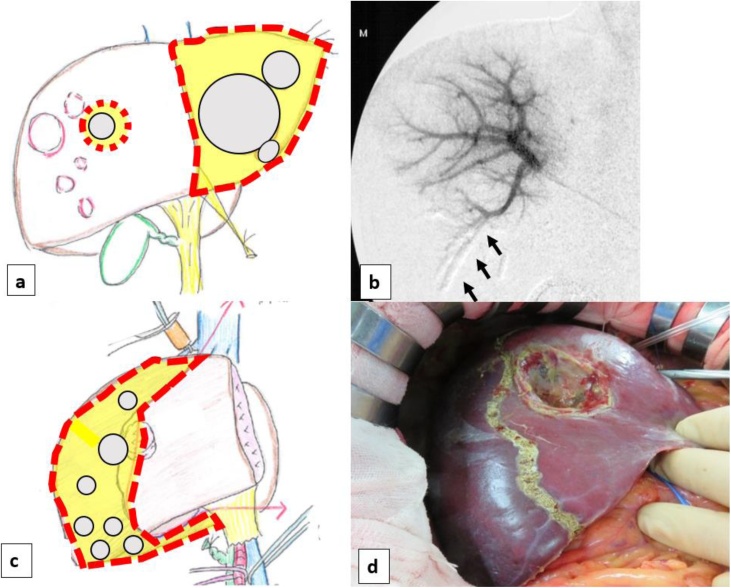
Diagram and operative findings. a, b) First operation (a) Diagram showing line of liver resection for the first operation. (b) Portal vein embolization of the posterior segmental branch (arrows). c, d) Second operation (c) Diagram showing line of liver resection for the second operation. (d) Intraoperative image shows the demarcation line appearing after clamping the right hepatic vein.

**Fig. 3:**
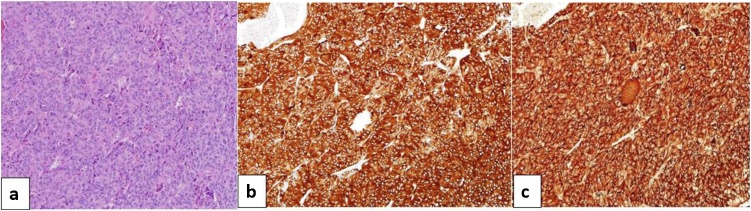
Pathological diagnosis. Microscopic examination of the resected liver specimen shows proliferation of atypical cells displaying features characteristic of atypical pulmonary carcinoid. (a) Hematoxylin & eosin staining ×200: The tumor comprises trabecular, palisading and rosette-like structures with round nuclei of various sizes and eosinophilic granular cytoplasm. (b) Immunohistological staining ×200: Immunochemistry of atypical carcinoid positive for synaptophysin. (c) Immunohistological staining ×200: Immunochemistry of atypical carcinoid positive for chromogranin A.

A 48-year-old man was referred to our department with multiple liver tumors detected on follow-up computed tomography (CT). He had undergone right upper lobectomy of the lung for AC 2 years previously. The pathological stage of the primary had been T2aN0M0 (stage IB). He was asymptomatic and had no relevant medical history. Contrast-enhanced CT and gadolinium ethoxybenzyl diethylenetriamine pentaacetic acid (Gd-EOB-DTPA)-enhanced magnetic resonance imaging (MRI) revealed multiple metastases in both lobes of the liver (Fig. 1a–d). These tumors were located in segments 2, 3, 5/8 and the right hepatic vein drainage area. Positron emission tomography (PET)-CT showed no extrahepatic tumor manifestations. No lymph node metastases or peritoneal dissemination were identified. The tumor markers carcinoembryonic antigen, pro-gastrin-releasing peptide, and protein induced by vitamin K absence or antagonist-II were all within normal limits. Results of preoperative liver function testing were unremarkable and indocyanine green retention rate at 15 min was 5% (normal). We planned complete resection of the metastases in both lobes of the liver using a two-stage hepatectomy, as CT volumetry of the future liver remnant volume (FLRV) showed 35 % of the total liver volume, which was marginal. During the first-stage, left lateral segmentectomy, partial hepatectomy of segment 5/8 and portal vein embolization of the posterior segmental branches through the ileocolic vein were performed concomitantly **(**Fig. 2a,b). Fibrin glue mixed with iodized oil was used as embolic material. The right lobe of the liver was partly mobilized to allow safe partial hepatectomy of segment 5/8. The Pringle’s maneuver was performed by tightening a rubber tube around the entire hepatoduodenal ligament. Four tumors were identified in the surgical specimen. CT on postoperative day 14 showed FLRV had increased to 45 % of the total liver volume, which was judged to be sufficient. The second-stage surgery was therefore performed 21 days after the first surgery, as resection of the right hepatic vein drainage area (i.e., posterior and dorsal section of the anterior segment) (Fig. 2c). During the second-stage surgery, adhesion was dissected and cholecystectomy was performed. The right lobe of the liver was then fully mobilized and the short hepatic veins draining into the vena cava were divided between ligation. The hepatoduodenal ligament was encircled and divided into the hepatic artery, portal vein and bile duct. The demarcation line was visualized by clamping the common hepatic artery and right hepatic vein (Fig. 2d). Hepatic transection was performed under the Pringle’s maneuver. The Glissonean pedicles of the anterior dorsal branches and posterior branch were separately ligated at the root. Finally, the root of the right hepatic vein was transected and the surgical specimen was removed. Eight tumors were identified in the surgical specimen. Histopathologically, all tumors comprised trabecular, palisading, or rosette-like structure with round nuclei of various sizes and eosinophilic granular cytoplasm. Tumor cells stained positive for immunohistochemical markers CD56, synaptophysin, and chromogranin A, with 2 mitoses per 10 high-power fields. The tumors were finally diagnosed as metastatic AC tumors originating from the lung **(**Fig. 3a–c). The patient developed postoperative bile leak, which was treated with endoscopic retrograde biliary drainage and percutaneous bile leakage drainage. He was discharged on postoperative day 82, and has since been followed without any adjuvant therapy. As of the time of writing, 24 months postoperatively, no tumor recurrence has been identified.

## 3 Discussion

The term ‘carcinoid’, speciﬁcally separated into typical carcinoid (TC) and AC, has been widely used in pulmonary disease, unlike digestive disease. At this stage, no move has been made toward World Health Organization terminologies for gastroenteropancreatic neuroendocrine tumors (GEP-NETs) [6].

AC shows a higher rate of distant metastases and a poorer prognosis. The 5- and 10- year survival rate for AC ranges from 40 % to 59 %, and from 35 % to 59 % respectively [1,2]. Metastatic disease has a much poorer 5-year survival rate, at 14–25 % [1,2]. AC with liver metastases is associated with the worst survival, compared with primaries in other organs [7].

Glazer reported that early and aggressive surgical management of liver metastases from neuroendocrine tumors is associated with significantly improved long-term survival rates. [7] Surgical resection of liver metastases can be considered with curative intent, to aid symptom control or for debulking when >90 % of tumor can be removed. According to the European Neuroendocrine Tumor Society (ENETS) consensus guidelines for the management of patients with GEP-NETS, the minimal requirements for curative intent are an absence of unresectable lymph node and extra-abdominal metastases and a lack of diffuse or unresectable peritoneal carcinomatosis [8]. Several patients showing bilobar multiple liver metastases at the time of diagnosis have been considered inoperable because of insufficient FLRV or unresectable peritoneal dissemination [8]. Accordingly, patients with inoperable liver metastases are often treated using non-surgical therapies such as somatostatin analogue, peptide receptor radionuclide therapy, transarterial embolization, radiofrequency ablation or chemotherapy for symptom control or mass reduction [1,2].

Two-stage hepatectomy is now an established strategy for patients with multiple bilobar colorectal liver metastases, due to the short- and long-term outcomes [3,4]. According to one recent review, postoperative complication rates after the first and second stages of surgery are 0–37 % and 11–60 %, and mortality rates are 0–4 % and 0–6 %, respectively [4]. Regarding long-term outcomes, the 5-year overall survival rate after completing two-stage hepatectomy range from 32 % to 64 %, with a median survival time of 22–44 months [4].

In the current case, sufficient FLRV was achieved by portal vein embolization of the posterior segmental branch during the first surgery, allowing subsequent complete hepatic resection to be performed.

Little evidence is available to guide surgery for liver metastases of AC, and therefore the present strategy was based on ENETS consensus guidelines for gastrointestinal neuroendocrine tumors [8,9].

Ligation of the posterior branches of the portal vein is complicated and may make the second-stage hepatectomy technically difficult [10], so we performed intravascular embolization from an ileocolic vein during the first-stage operation. In addition, efforts were made to minimize dissection during the first-stage surgery to prevent further fibrous adhesion.

Several cases of AC with recurrence beyond 10 years from the initial resection have been reported [[11], [12], [13]]. Long-term follow-up is therefore required in the current case.

## 4 Conclusions

We successfully treated a patient with multiple liver metastases of AC using a two-stage hepatectomy combined with portal vein embolization of the posterior segmental branches. Two-stage hepatectomy may be a good option for bilobar multiple liver metastases of AC.

## Declaration of Competing Interest

There are no conflicts of interest.

## Funding

We have no sponsors.

## Ethical approval

This study has been exempted by our institution.

## Consent

Written informed consent was obtained from the patient for publication of this case report and any accompanying images.

## Author contribution

TK: study design, data collection, data analysis, writing.

SO: critical revision

KY: final approval of the article

Any other authors: data collection

All authors read and approved the final manuscript.

## Registration of research studies

This paper is case report. The authors don’t need to register this work.

## Guarantor

Teppei Kamada, the corresponding author of this manuscript accept full responsibility for the work and the conduct of the study, access to the data and controlled the decision to publish.

## Provenance and peer review

Not commissioned, externally peer-reviewed.
